# The Outcomes and Adverse Drug Patterns of Immunomodulators and Thrombopoietin Receptor Agonists in Primary Immune Thrombocytopenia Egyptian Patients with Hemorrhage Comorbidity

**DOI:** 10.3390/ph16060868

**Published:** 2023-06-12

**Authors:** Eman Mostafa Hamed, Ahmed R. N. Ibrahim, Mohamed Hussein Meabed, Ahmed M. Khalaf, Doaa Mohamed El Demerdash, Marwa O. Elgendy, Haitham Saeed, Heba F. Salem, Hoda Rabea

**Affiliations:** 1 Department of Clinical Pharmacy, Faculty of Pharmacy, Nahda University (NUB), Beni-Suef 62521, Egypt; marwa.elgendy@nub.edu.eg; 2 Department of Clinical Pharmacy, College of Pharmacy, King Khalid University, Abha 61421, Saudi Arabia; aribrahim@kku.edu.sa; 3 Department of Pediatrics, Faculty of Medicine, Beni-Suef University, Beni-Suef 62521, Egypt; m1meabed2@med.bsu.edu.eg; 4 Department of Internal Medicine and Clinical Hematology, Beni-Suef University, Beni-Suef 62521, Egypt; dr.ahmed201176@yahoo.com; 5 Department of Internal Medicine and Clinical Hematology, Faculty of Medicine, Cairo University, Giza 54212, Egypt; dr_eldemerdash@kasralainy.edu.eg; 6 Department of Clinical Pharmacy, Teaching Hospitals of Faculty of Medicine, Faculty of Medicine, Beni-Suef University, Beni-Suef 62521, Egypt; 7 Clinical Pharmacy Department, Faculty of Pharmacy, Beni-Suef University, Beni-Suef 62521, Egypt; haitham.sd1@gmail.com (H.S.); hoda.ahmed@pharm.bsu.edu.eg (H.R.); 8 Department of Pharmaceutics and Industrial Pharmacy, Faculty of Pharmacy, Beni-Suef University, Beni-Suef 62521, Egypt; heba.salem@nub.edu.eg; 9 Pharmaceutics and Industrial Pharmacy Department, 6 October Technological University, Giza 62521, Egypt

**Keywords:** autoimmune disease, primary immune thrombocytopenia, primary immune thrombocytopenia, dexamethasone, prednisolone, azathioprine, rituximab, eltrombopag, Romiplostim

## Abstract

Immune thrombocytopenia (ITP) treatment has evolved recently. However, none of the treatments have only benefits without drawbacks. This study aimed to compare the clinical outcomes and adverse drug patterns of Eltrombopag, Romiplostim, Prednisolone + Azathioprine, High Dose-dexamethasone (HD-DXM) (control group), and Rituximab in primary ITP Egyptian patients. All patients were initiated with corticosteroids, HD-DXM, as a first-line treatment for the first month immediately following diagnosis. Four hundred sixty-seven ITP patients were randomly assigned to five groups. The outcome measures were judged at baseline, at the end of treatment (6 months), and after an additional 6-month free treatment period. The follow-up period for which relapse is noted was 6 months after the end of treatment. Eltrombopag and Romiplostim resulted in a significantly higher incidence of sustained response than Rituximab, HD-DXM, and Prednisolone + Azathioprine (55.2% and 50.6% vs. 29.2%, 29.1%, and 18%, respectively; *p*-value < 0.001). More patients on immunomodulators (Prednisolone+ Azathioprine, HD-DXM, and Rituximab) relapsed than those on Romiplostim and Eltrombopag (81.9%, 70.8%, and 70.7% vs. 49.3%, and 44.7%, respectively; *p*-value < 0.01). We also describe 23 reports of pulmonary hypertension with Prednisolone+ Azathioprine and 13 reports with HD-DXM. The thrombotic events occurred in 16.6% and 13% of patients who received Eltrombopag and Romiplostim treatment, respectively. Most patients had at least one or two risk factors (92.8% of cases). Corticosteroids are effective first-line therapy in primary ITP patients. However, relapse is frequent. Eltrombopag and Romiplostim are safer and more effective than Prednisolone, HD-DXM, and Rituximab. They might be reasonable beneficial options after a one-month HD-DXM regimen.

## 1. Introduction

Primary immune thrombocytopenia (ITP) is an autoimmune disorder that triggers immune-mediated platelet destruction and impaired megakaryocyte platelet production [1,2]. The severe complication of ITP is bleeding, which affects 60% of patients, with 6% experiencing severe bleeding and only 0.4% cerebral hemorrhage [3]. Approximately 80% of ITP patients are primary ITP. Lymphoma, systemic autoimmune disease, a persistent viral infection such as HIV or hepatitis B or C viruses (HCV), a fundamental immunological impairment, or medications are causes of secondary ITP [4].

The guidelines recommended Prednisolone (PSL) or High Dose-dexamethasone (HD-DXM) as the first-line regimen for primary ITP patients for a short term to avoid corticosteroid adverse events [5,6]. Second-line therapies such as Azathioprine (AZA), Rituximab (RTX), and thrombopoietin receptor agonists (TPO-RAs) are necessary for patients who become intolerant to corticosteroids or who relapse after splenectomy [7,8]. Eltrombopag (ELTRO) and Romiplostim (ROMP) are TPO-RAs that have been licensed in the management of primary ITP [7]. TPO-RAs elevate the PLTs count with comparatively minor adverse events. However, thrombosis is one of their probable adverse events of concern [9,10]. TPO-RAs have been compared to corticosteroid regimens or placebos in recent clinical trials [11,12]. 

Several studies remarked that combination therapy achieved high efficacy [13] but had more fatal bleeding incidents [14]. Further studies comparing second-line treatments as a monotherapy are recommended. 

A longitude randomized study assessed the long-term efficacy of immunomodulators and thrombopoietin receptor agonists in primary Egyptian ITP children, focusing on the efficacy of therapeutic regimens in sustaining the PLTs count without studying the adverse events of the different regimens [11]. The safety profile and long-term efficacy have been reported in the Western world. Conversely, studies from information-constraint backgrounds are limited in the developing world [15]. Consequently, it is frequently challenging for developing world physicians to make decisions based on limited evidence. A head-to-head comparison is desired to determine the most effective and safest approach for newly diagnosed primary ITP. We aimed to investigate the safety and efficacy of frontline corticosteroids and the most common second-line treatments following one month of HD-DXM in Egyptian primary ITP patients over the first 12 months of the diagnosis. 

## 2. Results

### 2.1. Demographic Data

**Figure 1:**
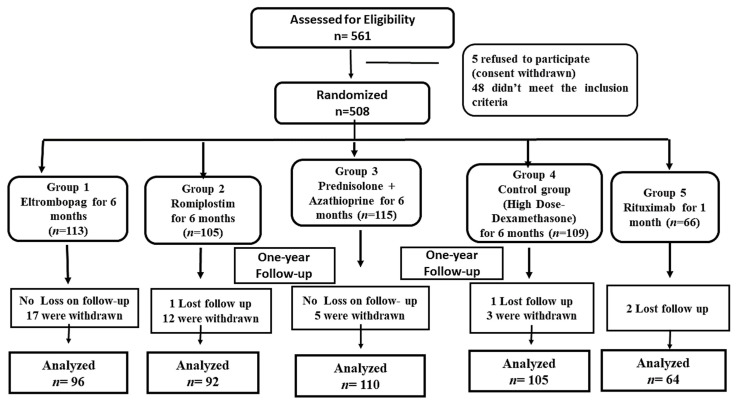
The Flow of the Study Randomized Allocation.

ITP subjects that fulfilled the inclusion criteria were conducted at the hematology outpatient clinic and assigned to one of five groups. Patients with thrombocytopenia associated with chemical-induced lupus (*n* = 15), immune thyroid diseases (*n* = 10), a lymphoproliferative disease (*n* = 2), or chronic infection, such as Helicobacter pylori (*n* = 5), human immunodeficiency virus (HIV) (*n* = 4) or hepatitis C virus (HCV) (*n* = 7) were excluded (*n* = 43). In addition, patients with pulmonary, cardiac, or renal dysfunction (*n* = 2); who had received NSAIDs or anti-platelets within one month from the initiation of the enrollment were also excluded (*n* = 3). Thirty-seven subjects withdrew during the study enrollment (due to non-compliance), and four subjects missed the follow-up, while 467 patients completed the randomized study (Figure 1). All the participants in the study were comparable and similar (*p* > 0.05) in terms of baseline clinical, hematological markers, and demographics, as presented in Table 1. Furthermore, all the baseline data (age or gender) did not display a statistically significant difference in the PLTs count in every studied group (*p* > 0.05). 

### 2.2. Effect of Immunomodulators and Thrombopoietin Receptor Agonists Therapy on Platelets Count

**Figure 2:**
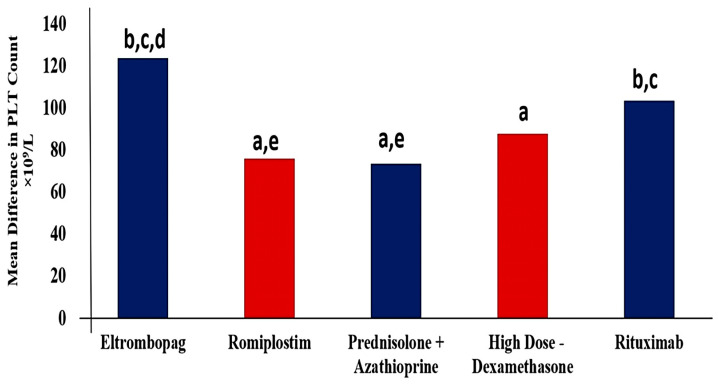
Mean difference in Platelets count between pre- and post-regimens in 467 subjects in each group after 6 months of therapy. ^a^ Significantly different from the Eltrombopag regimen at *p*-value < 0.05. ^b^ Significantly different from the Romiplostim regimen at *p*-value < 0.05. ^c^ Significantly different from the Prednisolone + Azathioprine regimen at *p*-value < 0.05. ^d^ Significantly different from the High Dose-Dexamethasone regimen at *p*-value < 0.05. ^e^ Significantly different from the Rituximab regimen at *p*-value < 0.05.

All groups demonstrated a significant beneficial effect of used regimens expressed as higher PLTs count after 6 months of starting the therapeutic regimens (Figure 2). Regarding the ELTRO group, the PLTs count was significantly increased by 87.1% from 18.2 ± 1.31 × 10^9^/L to 141.8 ± 10.8 × 10^9^/L (*p*-value < 0.001). The average PLTs count was 18.4 × 10^9^/L before ROMP therapy, and it significantly increased to 94.2 ± 6.9 by 80.4% (*p*-value < 0.001). PLTs count increased by 78.8% (from 19.7 to 93.2 × 10^9^/L) and by 82.4% (from 18.54 to 105.9 × 10^9^/L) after administration of PSL+AZA regimen and HD-DXM, respectively (*p*-value < 0.001). RTX significantly elevated the PLTs count to 125.2 × 10^9^/L from the baseline of 21.85 × 10^9^/L (*p*-value < 0.001). Patients who received ELTRO had a greater PLTs level than other medications, followed by RTX. While HD-DXM, ROMP, and PSL+AZA showed a lower efficacy than ELTRO and RTX, respectively. There is a statistical significance (*p*-value < 0.01) in PLTs count difference between ELTRO and HD-DXM, ROMP or PSL. While no statistical significance was found in HD-DXM group compared to RTX, PSL+AZA or ROMP, (*p*-value > 0.05). Besides these findings, there is a statistical difference between ROMP and PSL+AZA compared to RTX (*p*-value = 0.043 and 0.023, respectively). These observations are profiled in Table 2.

### 2.3. Response Results

The overall response was significantly higher in ELTRO and ROMP groups than in PSL+AZA and RTX (88.5% and 81.5% vs. 65.4%% and 64.1%, *p*-value < 0.01). 

Moreover, there was a significant difference between ELTRO and HD-DXM (88.5% vs. 75.2%, *p*-value = 0.01), while no significant difference was found between ROMP and HD-DXM (81.5%vs. 75.3%, *p*-value = 0.287).

There is a statistically significant difference in the prevalence of complete response in patients who received ELTRO and HD-DXM compared to PSL+AZA (60% and 51.8% vs. 36.1%, *p*-value = 0.003 and 0.049). CR was achieved with a higher percentage in patients who received ELTRO than RTX or ROMP; however, there was no significant difference (60% vs. 51.2% and 45.3%, *p*-value > 0.05). The sustained response achieved in patients who received ELTRO and ROMP was significantly greater than those in RTX, HD-DXM, and PSL+AZA (52.2 and 50.6% vs. 29.2%, 29.1% and 18%, respectively, *p*-value < 0.01) but no statistically significant difference between them *p*-value >0.05. Moreover, ELTRO and ROMP resulted in significantly fewer relapsed patients than RTX, HD-DXM, and PSL+AZA (44.7% and 49.3% vs. 70.7%, 70.8%, and 81.9%, respectively; *p*-value < 0.01). Besides, there was no significant difference between PSL+AZA, RTX, and HD-DXM in the number of ITP subjects who relapsed (*p* > 0.05). 

**Figure 3:**
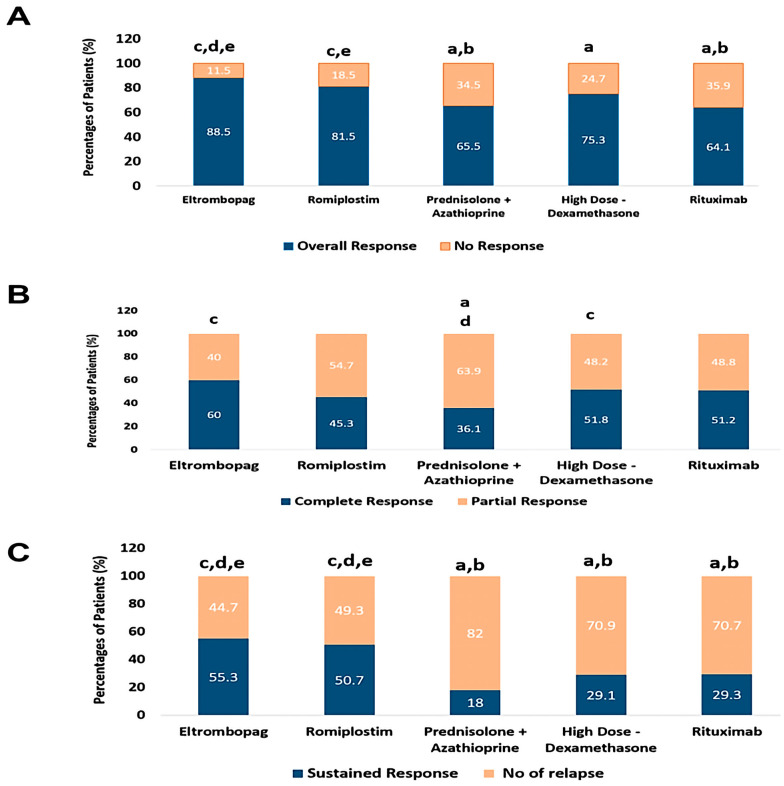
Comparison between immunomodulators and thrombopoietin receptor agonists according to percentages of patients’ response, (**A**); the percentages of patients who achieved the overall response rate (ORR) and no response (NR), (**B**); the percentages of patients who achieved the complete response (CR) and partial response (PR), (**C**); the percentages of patients who achieved the sustained response (SR) and Relapse. ^a^ Significantly different from the Eltrombopag regimen at *p*-value < 0.05. ^b^ Significantly different from the Romiplostim regimen at *p*-value < 0.05. ^c^ Significantly different from the Prednisolone + Azathioprine regimen at *p*-value < 0.05. ^d^ Significantly different from the High Dose-Dexamethasone regimen at *p*-value < 0.05. ^e^ Significantly different from the Rituximab regimen at *p*-value < 0.05.

The partial response was significantly higher in patients who received PSL+AZA than HD-DXM and ELTRO (63.6% vs. 48.1% and 40%, respectively; *p*-value < 0.01). Conversely, no statistical difference in PR was highlighted between ROMP and RTX with PSL+AZA, although the PR in PSL+AZA was higher than in ROMP and RTX (63.6% vs. 54.6% and 48.7%, *p*-value > 0.05). The patient’s failure of initial response to RTX, PSL+AZA, and HD-DXM was more significant than those in ELTRO and ROMP (35.9%, 34.5%, and 24.7% vs. 11.4 and 18.4%, respectively; *p*-value < 0.001). These findings are profiled in Table 2 and Figure 3.

### 2.4. Safety Assessment

Adverse events were virtually recorded in line with the pivotal laboratory estimation and clinician reports. The intervention outcomes and procedures were noted as well. Safety endpoints involved bleeding episodes, and laboratory results. Bleeding symptoms were rated concerning the World Health Organization bleeding scale and the ITP-specific hemorrhage evaluation tool [16]. AEs were rated based on the Common Terminology Criteria for AEs [17].

#### 2.4.1. Incidence and Outcome of Adverse Events during Thrombopoietin Receptor Agonists (ELTRO and ROMP) Therapy 

Sixteen thromboses cases happened with ELTRO and twelve during ROMP, involving arterial thrombosis and deep venous thrombosis that happened by both TPO-RAs. The whole thromboses were developed throughout the initial 10 months of the regimens. The PLTs count was between 186 and 390 × 10^9^/L during the issue. In 26 (92.8%) cases, at least one or two additional risk factors were present. Thrombocytosis was significantly noted in five (17.85%) cases (two on ELTRO and three on the ROMP regimen). There were 13 arterial thromboses: 5 ischemic strokes during both TPO-RAs, 6 myocardial infarctions on the ROMP regimen, and 2 myocardial infarctions during the ELTRO regimen. In eighty-one (84.3%) ITP subjects, transaminase values (AST, ALT) were two-fold elevated, but in one patient, a temporary upsurge in the ALT level up to 125 U/L and AST level up to 187 U/L was documented during ELTRO regimen.

#### 2.4.2. Incidence and Outcome of Adverse Events during Immunomodulators (PSL+AZA, HD-DXM, and RTX)

The most notable AEs during corticosteroids is bleeding. The epistaxis had occurred more frequently with corticosteroids than with ROMP, ELTRO, and RTX groups. Bleeding was more successfully controlled in the ELTRO group, with fewer bleeding episodes than HD-DXM (*p*-value = 0.004). Additionally, Bleeding episodes in the ELTRO, ROMP, and RTX therapy were significantly minor incidents than during the HD-DXM and PSL+AZA regimen through 12 months (6.2%, 10.8% and 14% vs. 59% and 78.1%, *p*-value < 0.001). Similar significance was also observed in higher bleeding episodes in PSL+AZA than HD-DXM (78.1% vs. 59%, *p*-value < 0.01). In contrast, there was no statistically significant difference in the incidence of bleeding events between ELTRO, ROMP, and RTX (all *p*-value > 0.05). The incidence of osteoporosis was most frequent among patients who received HD-DXM and PSL+AZA regimen (93.3% and 84.5%, respectively).

One of the unexpected findings was that one patient acquired lymphoma during azathioprine treatment. There were 27 (42.1%) reported infections in the RTX group, including upper respiratory tract (12 patients), tropical (three patients with fascioliasis and one patient with typhoid), urinary tract infection (7 patients), pneumonia (3 patients), and Herpes genitalis (1 patient). Nine cases of deep vein thrombosis developed during RTX therapy. Two deaths occurred during the treatment period: one in the ELTRO group from cardiac arrest and one from intracranial hemorrhage in the prednisolone + azathioprine group. No fatalities were thought to be connected to treatment. All these adverse events are profiled in Table 3.

### 2.5. Study limitations 

Extensive essential pharmaco-epidemiological studies are required to compare the adverse events of the two TPO-RAs and the three immune modulators with more investigations. Future studies might include further analyses with targeted outcomes of interest. 

## 3. Discussion

Despite the emerging TPO-RAs, conservative immune modulators are still a cornerstone first-line for the management of ITP. Conventional first-line ITP therapies can induce a fast PLTs response; however, this response is frequently transient. This study compared the efficacy and safety of ELTRO, ROMP, PSL+AZA, HD-DXM, andRTX. All indicators of successful ITP therapy, including the increase in PLTs count, lower bleeding events, decreased need for rescue drugs, and lower platelets transfusion, were assessed in the five groups. 

The ORR to ELTRO was 88.5%, with CR and PR as 51 (60%) and 34 (40%), respectively, which was higher than ORR, 54.3%, CR, 48.6%, and PR, 5.7%, respectively, in Qi Liu and Kundan Mishra’s clinical study [15,18]. The average interval times needed for PLTs to reach 100 × 10^9^/L were 16 (6–38) and 20 (15–64) days, respectively, which was consistent with earlier trials [18]. An Italian multi-center retrospective study was closely approximate with that finding, presenting that the ORR to ELTRO was 94.2% [19]. 

The SR achieved in patients who received ELTRO, and ROMP was significantly greater than those in RTX, HD-DXM, and PSL+AZA (52.2% and 50.6% vs. 29.2%, 29.1%, and 18%, respectively, *p*-value < 0.01). The SR was higher in the ELTRO than in the ROMP patients, although the difference was not significant statistically *p*-value = 0.558. Between the two TPO-RAs, no variations in response sustainability were identified. Those findings are in line with a systemic assessment of five controlled studies that investigated the effectiveness and safety of ELTRO and ROMP in primary ITP, and no distinction between the two types of treatment was found [20]. Patients on TPO-RAs had a much higher SR than those on immune modulators. More patients on immune modulators (PSL, HD-DXM, and RTX) relapsed than those on TPO-RAs (ROMP and ELTRO) (81.9%, 70.8%, and 70.7%, vs. 49.3% and 44.7%, respectively; *p*-value < 0.01). The low sustainability of remission was documented previously in a multi-center retrospective study [11]. The number of females who had SR was significantly higher than that of males among patients receiving ELTRO therapy (76.5% vs. 23.5%, *p*-value < 0.01). In a Chinese retrospective study, a better outcome for ELTRO was depicted among females rather than male ITP patients (75% vs. 25%, *p*-value < 0.01) [21].

The HD-DXM group had significantly better CR than PSL+AZA (51.8% vs. 36.1%; *p*-value = 0.049). These results are higher than those conducted by Yu Wei et al., who found that HD-DXM resulted in CR (50.5% vs. 26.8%; *p*-value = 0.001) compared to PSL [22]. A meta-analysis study reported a higher CR for HD-DXM parallel to the current study [23]. Retrospective clinical studies comparing the two regimens in small-scale cohorts produced similar outcomes [24,25,26], but other studies yielded conflicting results [27,28]. The number of females who had SR was significantly higher than that of males among patients receiving Immunomodulators (HD-DXM and PSL+AZA, 78.3% and 69.3% vs. 21.7% and 30.7%, respectively, *p*-value < 0.01). A cross-sectional study by James B. Bussel et al. obtained similar results and declared that females had a higher impact on the durable response than males (61% vs. 17%, *p*-value = 0.0078) [29]. In contrast, a previous study found that PLTs count and gender were not significantly correlated with remission rates of ITP following 12 or 24 months of follow-up [30].

The CR to RTX was around 51.2%; however, only 29.3% of patients sustained remission, and 70.7% relapsed after a long duration of response. The number of males who had SR was significantly higher than that of females among patients receiving RTX therapy (83.4% vs. 16.6%, *p*-value = 0.01). This is in line with previous studies screening the female majority in chronic ITP patients, giving weight to the idea that one of the risk factors for ITP chronicity is the female gender [11,31]. These results do not support the concept that there is no association between treatment outcomes with age or gender [32]. Remarkably, RTX is still one of the most predominant options for ITP therapy, even though studies indicate that RTX has underwhelming long-term response rates [33]. Ayat et al. highlighted such a high relapse rate and reported an initial 54% response rate to RTX, but the SR was 15% only [11]. Systematic meta-analyses network and reviews have confirmed the parallel impact of ELTRO and ROMP and the superior effectiveness of both drugs compared with RTX [34]. It was hypothesized that relapse is referred to the return of B cells to greater levels than those in subjects who never relapsed [35]. Combining belimumab, a B cell inhibitor, with RTX enhanced the ORR to RTX after one year in chronic ITP patients [36]. Unfortunately, belimumab has not been available in our Egyptian markets. 

ELTRO markedly decreased the bleeding events and PLTs transfusion more than RTX, ROMP, and PSL+AZA. The bleeding risk was more frequent in PSL+AZA than in HD-DXM (78.1% vs. 59%) patients through the first year of ITP diagnosis. Another multi-center study has confirmed these findings [25]. 

All therapeutic regimens had potential adverse events and complications. This study reported twelve thrombotic episodes with ROMP and sixteen with ELTRO. Most of those patients had at least one cardiovascular risk factor (diabetes, hypertension, or smoking), except two patients. This result is consistent with previous studies [37,38,39]. A recently updated systematic review revealed similar findings [10]. Despite this, ITP may elevate the risk of thrombosis [40]. The possible explanation of thrombosis was that TPO-RA increases the PLTs count and encourages the synthesis of active PLTs and new micro-particles [9,10]. A sudden increase in PLT may trigger thrombotic episodes [41]. In the previous study by Moulis, G. et al., twelve venous thromboses were reported during ROMP use and seven with ELTRO in addition to the superior sinus occlusion that happened with both TPO-RAs [42]. An integrated -analysis (*n* = 921) evidenced that ROMP therapy was associated with thrombotic episodes [43]. Moreover, 2% of patients developed liver cirrhosis on using ELTRO, despite that those patients had no history of HCV infection or hepatic dysfunction. Over 95% of patients had plantar fasciitis with ELTRO. The patients have been referred to orthopedic surgeons who confirmed the diagnosis of plantar fasciitis. This AE had not occurred with ROMP or immunomodulators. This current study is the first study to mention this point which needs further investigation. 

The ROMP patients had fewer serious AEs and better improvements in the subject health than the immunomodulators. One of the most often observed findings was that ROMP and ELTRO were associated with a higher risk of hematological AEs. Twenty-three cases of leukopenia and seventeen cases of anisocytosis were reported with ELTRO and none with ROMP. Thirty-three leukocytosis cases and eleven lymphocytosis cases are counted with ROMP and none with ELTRO. In contrast, the previous studies documented that a higher hematological AEs risk was reported with ROMP and none with ELTRO. Similarly, one previous study demonstrated prompt of red or white cells bone marrow progenitors only during ROMP in the French pharmacovigilance database [42]. 

The current study reported that corticosteroids have high initial effects, but patients frequently suffer severe adverse events such as hyperglycemia, hypertension, pulmonary hypertension, heart failure, and osteoporosis [44,45]. The incidence of osteoporosis was most frequent among patients who received HD-DXM and PSL+AZA regimen (93.3% and 84.5%, respectively). Previous studies documented that adults receiving corticosteroids a long term developed bone fractures and osteoporosis [44,46,47]. Therefore, recent studies recommended use of Bisphosphonates as prophylaxis against corticosteroids-induced osteoporosis in elderly patients with primary immune thrombocytopenia [48,49]. One of the surprising results is that fourteen ITP patients acquired aplastic anemia during corticosteroid therapy which was controlled with allogeneic hematopoietic stem cell transplantation (HSCT) [50]. This could be attributed to COVID-19 infections or vaccine schedules [51].

The present study reported 16.1% and 11.8% of infection cases during HD-DXM and PSL+AZA. We could explain infection related to low PLTs count, response rates, and longer hospitalization duration during PSL+AZA in primary ITP patients. This might be due to low immunity [52]. Previous studies suggested that a short duration of corticosteroids could reduce the AEs [25]. A significantly increased of infection rate has also been reported during RTX therapy. In accordance with the previous reports revealed that the incidence of infections was due to a significant decrease in IgM following RTX treatment [53].

TPO-RAs showed a beneficial effect in this study. However, recent studies provided that TPO-RAs’ efficacy in the first-line setting is not supported by sufficient evidence [22,54,55,56]. These studies supported that TPO-RAs plus dexamethasone was shown to be a potential first-line treatment for ITP. On the other hand, a number of studies have been published with evidence for the effect of TPO-RAs therapy in the second-line setting [57,58,59]. 

Finally, AEs triggered by steroids often outweigh the advantages due to long-term course. AEs or inadequate response was the main reason that led medical professionals to think about switching the TPO-RA. ELTRO not only decreased the bleeding events and platelets transfusion more effectively than the other second lines regimen but also reduced severe disease conditions such as hyperglycemia, hypertension, and pulmonary hypertension.

## 4. Material and Methods

### 4.1. Patients Selection

Egyptian ITP patients who visited hospitals’ outpatient clinics were recruited under the supervision of experienced hematologists. This controlled multi-center prospective study was conducted in tertiary hospitals affiliated with hematology departments (Al-Kasr El-Einiy, Beni-Suef University, and Beni-Suef Health Insurance Hospital). Ethical approval was obtained on March, 2020 with recording number REC-H-PhBSU-22016 from the research ethics committee of the faculty of pharmacy, Beni-Suef University in Egypt. All patients provided a written statement of informed consent before participation in the study. All enrolled patients had an initial peripheral PLTs count of less than 30 × 10^9^/L or bleeding manifestations at the onset of enrollment. ITP was diagnosed primarily by excluding the other causes of isolated thrombocytopenia, such as cancers, chronic infection, such as Helicobacter pylori, human immunodeficiency virus (HIV) or hepatitis C virus (HCV), lupus, and drugs. The diagnosis was performed through physical examination, complete patient history, blood count, and study of the peripheral blood film to exclude further hematological issues, such as inherited thrombocytopenia and false thrombocytopenia [7]. Moreover, a bone marrow examination was performed when indicated [2]. Clinical investigations were commenced for the patients every month. The serum levels of ALT, AST, creatinine, and complete blood picture were measured in all subjects to certify their safety.

#### 4.1.1. Inclusion Criteria

Inclusion criteria were adult patients aged 18 years or older, diagnosed with primary ITP after excluding secondary causes and with an initial PLTs count of less than 30 × 10^9^/L or with hemorrhage manifestations. 

#### 4.1.2. Exclusion Criteria

Patients with a confirmed secondary ITP diagnosis such as chemicals-induced, systemic lupus erythematosus, immune thyroid diseases, a lymphoproliferative disease, or chronic infection, such as Helicobacter pylori, human immunodeficiency virus (HIV) or hepatitis C virus (HCV); with cardiac, renal, or liver disease; who had received NSAIDs or anti-platelets within one month before the initiation of the enrollment were excluded from the study. 

### 4.2. Study Design 

A prospective controlled randomized study was conducted on 467 primary ITP patients (371 females). The main objective of the study was to evaluate the efficacy and adverse events profile of the different therapeutic approaches during ITP. Upon the confirmation of the ITP diagnosis, all patients were immediately initiated with the High Dose-dexamethasone as a frontline therapy for ITP with a dose of 40 mg/m^2^ daily for 4 days in the first month for one cycle [6,24]. Then, the recruited patients who fulfilled the inclusion criteria were randomly assigned into one of five groups. Among these patients, the Eltrombopag group received 50 mg of Eltrombopag four hours before or after meals as oral daily doses for 6 months. The Romiplostim group received 3 μg/kg subcutaneous injection of Romiplostim once a week for 6 months [60]. The Prednisolone + Azathioprine group received 20 mg of Prednisolone three times daily and 1 mg/kg of oral Azathioprine once daily for two weeks, then tapering the Prednisolone dose through the subsequent weeks (6 weeks) [6,11]. While continuing treatment with Azathioprine for a total of six months. The control group received IV pulse (HD-DXM) therapy with 40 mg/m^2^ daily for 4 successive days in a 28-day cycle to complete the six cycles [24]. The Rituximab group received 500 mg/m^2^ intravenously of Rituximab once weekly for one month [61]. The first evaluation date of confirmed ITP diagnosis was well-defined as the first index date (baseline). After that, every patient visited the investigational site as the protocol prescribes once weekly to assess and adjust the doses of study medications. The outcome measures were judged at baseline, at the end of treatment (6 months), and after an additional 6-month free treatment period. 

### 4.3. Primary Outcome Measures 

The primary outcomes were the total percentage of patients achieving a sustained response (SR) till the end of the study, complete response (CR), and partial response (PR). CR was characterized by the absence of bleeding and an increase in the platelets count to above 100 × 10^9^/L after one month of the treatment. SR was defined as achieving CR or partial response (PR) until the end of the study with a 2-fold upsurge from starting point [62,63]. PR was represented as PLTs count ≥ 30 × 10^9^/L after one month following therapy, and no response (NR) was defined as platelets < 30 × 10^9^/L or bleeding [11]. The percentage of subjects who showed a complete or partial response to therapy is represented the overall response rate (ORR). Percentages of SR and relapsed patients were calculated from the total number of patients who exhibited overall response. The efficacy outcome was also assessed by comparing the frequency of patient bleeding episodes. The durable response was identified as sustaining PLTs count over 50 × 10^9^/L for an additional 6 months without extra ITP regimens, and this was the primary measure outcome.

### 4.4. Secondary Outcome Measures 

The secondary outcome measures were a number of patients relapsed and adverse events (AEs). Relapse was pointed out as PLTs count below 30 × 10^9^/L or bleeding episodes owing to thrombocytopenia afterward achieving the CR [11,62]. A team of a hematologist and a clinical pharmacist recorded physical examination findings and patients’ symptoms during clinic visits to assess safety profiles. Patients who experienced severe AEs have discontinued the medications, been closely monitored, or received emergency care. The pivotal adverse events were registered in the pharmacovigilance of the Egyptian Drug Authority with the number 11-332-024-645.

### 4.5. Statistical Analyses

The likelihood of a normal distribution for the continuous variables was examined. For numerical variables that were normally distributed, the mean and standard error were provided. For skew-distributed data, the median was provided. Results were analyzed descriptively, stratified by treatment regimens (Eltrombopag, Romiplostim, Prednisolone + Azathioprine, High Dose-dexamethasone (HD-DXM) (control group), and Rituximab). For most measures, chi-square analyses were performed to compare differences between each group with the other. A paired *t*-test was used to compare the PLTs count difference between pre-and post-therapeutic regimens. One-way ANOVA test was used to detect the significance of the difference in PLTs count, followed by LSD post hoc analysis for pairwise analysis. Mann–Whitney U tests were used to compare numerical samples. The outcomes were assessed at the 0.05 *p* level. The evaluation used the Statistical Package for the Social Sciences (SPSS) for Windows version 21.0 (SPSS, Chicago, IL, USA).

## 5. Conclusions

The pooled full analysis displayed a variance in durable responses in patients but a greater initial PLTs count response with few additional adverse events, which should be considered. HD-DXM and RTX achieved a high initial response, but the sustainability of the response was challenging for a long time. A higher relapsing rate occurred in patients who received PSL+AZA, HD-DXM, and RTX. The ELTRO subjects had a higher durable response, fewer platelets transfused, and a lower rate of bleeding episodes than ROMP and immunomodulators. ELTRO could be used following one month of HD-DXM as an emerging option for confirmed diagnosed ITP patients.

This study reported that severe adverse events had occurred during PSL+AZA and HD-DXM, such as pulmonary hypertension, left ventricle dysfunction, and hyperglycemia. Neurologic Adverse events were found with ROMP and low Adverse events with RTX. By sharing these adverse events profiles of regimens, clinicians will become more aware of the potentially severe adverse events of different regimens and closely monitor patients in the future. Further studies on adverse drug patterns, especially plantar fasciitis complications during ELTRO, require more investigations to find more evidence in future studies.

## Figures and Tables

**Table 1:** Demographic and baseline characteristics in the five study groups.

Characteristics	Eltrombopag (*n* = 96)	Romiplostim (*n* = 92)	Prednisolone+ Azathioprine (*n* = 110)	High_Dose-Dexamethasone (*n* = 105)	Rituximab (*n* = 64)	*p*-Value
Age (years), Median (range)	34.5 (18–62)	32.5 (18–62)	28 (18–65)	29.5 (18–65)	34.5 (18–58)	0.095
Sex, n (%): Male Female	18 (18.8%) 78 (81.3%)	30 (32.6%) 62 (67.4%)	18 (17.4%) 92 (83.6%)	15 (14.3%) 90 (85.7%)	15 (23.4%) 49 (76.5%)	0.601 0.182
Initial Platelet count (×10^9^/L), Mean ± SE	18.2 ± 1.31	18.4 ± 1.61	19.7 ± 1.73	18.54 ± 1.49	21.85 ± 1.49	0.580
Creatinine (mg/dL), Mean ± SE	0.95 ± 0.06	1.2 ± 0.01	1.1 ± 0.021	0.99 ± 0.05	1.12 ± 0.013	0.993
Serum ALT (IU/L), Mean ± SE	28.01 ± 1.8	30.09 ± 2.3	28.9 ± 1.51	30.02 ± 1.76	32.07 ± 1.93	0.211
Serum AST (IU/L), Mean ± SE	30.06 ± 2.1	29.5 ± 2.4	29.3 ± 1.1	29.01 ± 1.8	28.06 ± 2.5	0.455

**Table 2:** Comparison between Difference in Platelets Count (×10^9^/L) and Responses Following Immunomodulators and Thrombopoietin receptor agonists in Primary Immune Thrombocytopenia.

Variable	Eltrombopag (*n* = 96)	Romiplostim (*n* = 92)	Prednisolone+ Azathioprine (*n* = 110)	High Dose-Dexamethasone (*n* = 105)	Rituximab (*n* = 64)	*p*-Value
**Platelets_count (×10^9^/L)_pre-therapy, Mean ± SE**	18.2 ± 1.31	18.4 ± 1.61	19.7 ± 1.73	18.54 ± 1.49	21.85 ± 1.49	0.580
**Platelets_count Post-therapy (×10^9^/L), Mean ± SE**	141.8 ± 10.8 ^b,c,d^	94.2 ± 6.9 ^a, e^	93.2 ± 7.2 ^a, e^	105.9 ± 6.6 ^a^	125.2 ± 13.2 ^b,c^	<0.001 *
**Mean Difference in PLTs count, Mean ± SE**	123.5 ± 10.8 ^b,c,d^	75.8 ± 6.9 ^a, e^	73.5 ± 7 ^a, e^	87.3 ± 6.5 ^a^	103.3 ± 12.8 ^b,c^	0.024 *
**Overall Response, n (%)** **No of response, n (%)**	85/96 (88.5%) ^c,d,e^ 11/96 (11.5%) ^c,d,e^	75/92 (81.5%) ^c,e^ 17/92 (18.5%) ^c,e^	72/110 (65.5%) ^a,b^ 38/110 (34.5%) ^a,b^	79/105 (75.3%) ^a^ 26/105 (24.7%) ^a^	41/64 (64.1%) ^a,b^ 23/64 (35.9%) ^a,b^	0.001 * 0.001 *
**Complete response, n (%)** **Partial response,** **n (%)**	51/85 (60%) ^C^ 34/85 (40%) ^c^	34/75 (45.3%) 41/75 (54.7%)	26/72 (36.1%) ^a,d^ 46/72 (63.9%) ^a,d^	41/79 (51.8%) ^C^ 38/79 (48.2%) ^C^	21/41 (51.2%) 20/41 (48.8%)	0.047 * 0.047 *
**Sustained Response, n (%)** **Relapsed patients, n (%)**	47/85 (55.3%) ^c,d,e^ 38/85 (44.7%) ^c,d,e^	38/75 (50.7%) ^c,d,e^ 37/75 (49.3%) ^c,d,e^	13/72 (18%) ^a,b^ 59/72 (82%) ^a,b^	23/79 (29.1%) ^a,b^ 56/79 (70.9%) ^a,b^	12/41 (29.3%) ^a,b^ 29/41 (70.7%) ^a,b^	0.001* 0.001 *
**Need to Platelets Transfusion;** **n (%)**	4 (4.1%)	9 (9.7%)	67 (60.9%)	49 (46.4%)	8 (12.5%)	<0.01 *
**Need to Rescue Treatments;** **n (%)**	2 (2.08%)	14 (15.2%)	19 (17.2%)	15 (14.2%)	1 (1.5%)	<0.01 *

The overall response percentages were calculated from the total number of patients in each group. The sustained and relapsed percentages were calculated from the patients who achieved the overall response. The complete and partial response were calculated from the patients who achieved the overall response. * *p*-values ≤ 5% represent the comparison between five different groups. ^a^ Significantly different from the Eltrombopag regimen at *p*-value < 0.05. ^b^ Significantly different from the Romiplostim regimen at *p*-value < 0.05. ^c^ Significantly different from the Prednisolone + Azathioprine regimen at *p*-value < 0.05. ^d^ Significantly different from the High Dose-Dexamethasone regimen at *p*-value < 0.05. ^e^ Significantly different from the Rituximab regimen at *p*-value < 0.05.

**Table 3:** The Adverse Drug Patterns During the Immunomodulators and Thrombopoietin receptor agonists among Immune Thrombocytopenia Patients.

Variable	Eltrombopag (*n* = 96)	Romiplostim (*n* = 92)	Prednisolone+ Azathioprine (*n* = 110)	High Dose-Dexamethasone (*n* = 105)	Rituximab (*n* = 64)	*p*-Value
Headache, *n* (%)	48 (50%)	78 (84%)	34 (30%)	41 (39%)	20 (31.2%)	0.001
Bleeding-related episodes, *n* (%)	6 (6.2%)	23 (25%)	86 (78.1%)	62 (59%)	9 (14%)	0.001
Epistaxis, *n* (%)	3 (3.1%)	9 (9.7%)	69 (62.7%)	49 (46.6%)	6 (9.3%)	0.001
Gum bleeding, *n* (%)	5 (5.2%)	7 (7.9%)	61 (55.4%)	53 (50.4%)	14 (21.8)	0.001
Ecchymosis, *n* (%)	20 (20.8%)	43 (46.7%)	47 (42.7%)	58 (55.2%)	12 (18.75%)	0.001
Osteoporosis_and Bone fracture, *n* (%)	12 (12.6%)	41 (44.5%)	93 (84.5%)	98 (93.3%)	5 (7.6%)	0.001
Elevated_blood pressure, *n* (%)	7 (7.29%)	3 (3.26%)	65 (59.09%)	49 (46.66%)	2 (3.12%)	0.001
Thrombosis, *n* (%)	16 (16.6%)	12 (13%)	0 (0%)	0 (0%)	9 (14.06%)	0.001
Liver cirrhosis, *n* (%)	2 (2%)	1 (1%)	0 (0%)	0 (0%)	0 (0%)	0.001
Elevated_liver enzymes, *n* (%)	81 (84.3%)	5 (5.4%)	8 (7.2%)	3 (2%)	7 (10.9)	0.001
Peptic ulcer, *n* (%)	0 (0%)	0 (0%)	63 (57.2%)	19 (18%)	0 (0%)	0.001
Infection, *n* (%)	1 (1%)	0 (0%)	13 (11.8%)	17 (16.1%)	27 (42.1%)	0.001
Pulmonary hypertension, *n* (%)	6 (6.25%)	2 (2.17%)	23 (20.9%)	13 (12.3%)	2 (3.1%)	0.001
Left_ventricle dysfunction, *n* (%)	2 (2%)	0 (0%)	7 (6.3%)	4 (3%)	0 (0%)	0.001
Hyperglycemia, *n* (%)	2 (2%)	1 (1%)	46 (41.8%)	19 (18%)	1 (1%)	0.001
Hair loss, *n* (%)	73 (76%)	42 (45%)	59 (53.6%)	48 (45.7%)	26 (40.6%)	0.001
Aplastic Anemia	0 (0%)	0 (0%)	9 (8.1%)	5 (4.7%)	0 (0%)	<0.01
Plantar Fasciitis	92 (95.8%)	0 (0%)	0 (0%)	0 (0%)	0 (0%)	<0.001
Numbness or Tingling, *n* (%)	77 (80.2%)	65 (70.6%)	97 (88.1%)	91 (86.8%)	15 (23.4%)	0.001
Chronic_Anemia, *n* (%)	6 (6.2%)	54 (87%)	69 (62.7)	76 (72.3%)	11 (17.1%)	0.105
Dizziness or Fatigue, *n* (%)	6 (6.25%)	56 (60.8)	74 (67.2%)	81 (77%)	2 (3.1%)	0.180
Acne, *n* (%)	0 (0%)	0 (0%)	76 (69%)	89 (84.7%)	0 (0%)	0.001
Weight gain, *n* (%)	1 (1%)	1 (1%)	91 (82.7%)	98 (93.3%)	2 (3.1%)	0.03
Depression, *n* (%)	7 (7.2%)	79 (85.8%)	93 (84.5%)	99 (94.2%)	11 (17.1%)	0.001
Anxiety or insomnia, *n* (%)	17 (17.7%)	87 (94.5%)	86 (78.1%)	94 (89.5%)	5 (7.8%)	0.001

## Data Availability

Data are contained within the article.
